# Chicken Coccidiosis: From the Parasite Lifecycle to Control of the Disease

**DOI:** 10.3389/fvets.2021.787653

**Published:** 2021-12-21

**Authors:** Carolina Mesa-Pineda, Jeffer L. Navarro-Ruíz, Sara López-Osorio, Jenny J. Chaparro-Gutiérrez, Luis M. Gómez-Osorio

**Affiliations:** ^1^Nutri-Solla Research Group, Solla S.A., Itagüí, Colombia; ^2^CIBAV Research Group, Facultad de Ciencias Agrarias, Universidad de Antioquia, Medellín, Colombia; ^3^Alura Animal Health and Nutrition, Medellín, Colombia

**Keywords:** chickens, *Eimeria*, oocysts, anticoccidials, diagnostic

## Abstract

The poultry industry is one of the main providers of protein for the world's population, but it faces great challenges including coccidiosis, one of the diseases with the most impact on productive performance. Coccidiosis is caused by protozoan parasites of the genus *Eimeria*, which are a group of monoxenous obligate intracellular parasites. Seven species of this genus can affect chickens (*Gallus gallus)*, each with different pathogenic characteristics and targeting a specific intestinal location. *Eimeria* alters the function of the intestinal tract, generating deficiencies in the absorption of nutrients and lowering productive performance, leading to economic losses. The objective of this manuscript is to review basic concepts of coccidiosis, the different *Eimeria* species that infect chickens, their life cycle, and the most sustainable and holistic methods available to control the disease.

## Introduction

The poultry industry is one of the main suppliers of animal protein worldwide, contributing both meat and eggs (1, 2). This is an industry in constant growth, as demonstrated by the United States Department of Agriculture (USDA). The USDA reported that 102.9 million tons of chicken meat were produced in January of 2020, which represents a 3.9% increase compared to the same period of the previous year (3). This increase is important, given that by 2050 the human population is expected to exceed nine billion people, making sustainable and safe protein production a worldwide priority (4). Any pathogen that compromises the efficiency of a poultry production system can pose a threat to food security worldwide (5).

There are many pathogens of great importance in the poultry industry, and among these are several coccidiosis-causing species of *Eimeria* belonging to the Apicomplexa phylum. These are obligate intracellular parasites with special organelles within the apical complex. These organelles are necessary for invasion of the host's intestinal cells (6). There are seven species of *Eimeria* recognized in poultry, each of them targeting a specific niche within the intestines and each with different pathogenicity characteristics (7).

The infection process begins with the ingestion of sporulated oocysts (their infectious form); depending on the species, infection can cause deficiencies in the absorption of nutrients, reduction in growth rates and, in the case of the most pathogenic species, increased mortality (8). Coccidiosis control has focused on several strategies including: management practices at the farm level, vaccines, and natural and traditional anticoccidials (9), the latter being the most successful and frequently used method of control (10). However, heavy use of anticoccidials has selected for strains of resistant parasites (11, 12). As a result of drug resistance, diminished performance, and increased mortality, coccidiosis is one of the most economically important diseases of poultry (13).

The objective of this manuscript is to review basic concepts of coccidiosis, including the different *Eimerian* species that infect chickens, their life cycle and the most sustainable and holistic methods to control the disease. The current global trend toward reduced use of anticoccidial drugs in poultry production requires us to improve our understanding of the causative agent and the pathogenesis of the disease in order to achieve better control.

## Etiological Agent of Avian Coccidiosis

Coccidia consist of a wide variety of unicellular parasites in the protozoan subgroup of the phylum Apicomplexa. As a group, coccidia of the genus *Eimeria* (*Eimeridae* family) are species-specific, infecting a single host species or a group of closely related hosts (14). The phylum (Apicomplexa) is characterized by obligate intracellular parasites, which possess unique specialized organelles that form the apical complex (15). These include: micronemes, rhoptries, dense granules, and conoid and polar rings (Figure 1.1) that provide the structural stability required during invasion of the host cell (15). Infection by a sufficiently large number of coccidia produces clinical manifestations of the disease called “coccidiosis,” whereas subclinical infections are asymptomatic but cause adverse effects on performance. The mildest form of infection that causes no symptoms and no adverse effects on performance infection is called “coccidiasis” (16–18). *Eimeria* spp. destroy host mucosal cells (19) as they invade enterocytes to begin their multi-stage replication process. This results in pathological changes such as elevated cell permeability, nutrient and plasma protein leakage, and impaired digestion and protein absorption (20, 21). Additionally, it causes morphologic alteration in the intestinal mucosa resulting in the reduction of absorptive surface area (20), compromising chicken well-being and productivity (21). The coccidia cycle is short, with an approximate duration of 4–6 days, depending on the species. The mode of transmission is oral-fecal and the infection can easily be transmitted through ingestion of sporulated oocysts (infective state of the parasite). Once inside the microenvironment of the host's intestinal tract, where they are exposed to digestive enzymes, the oocysts undergo excystation in the gizzard, aided by mechanical disruption, ultimately releasing sporozoites (6, 20) which start the life cycle of the coccidian (22).

**Figure 1:**
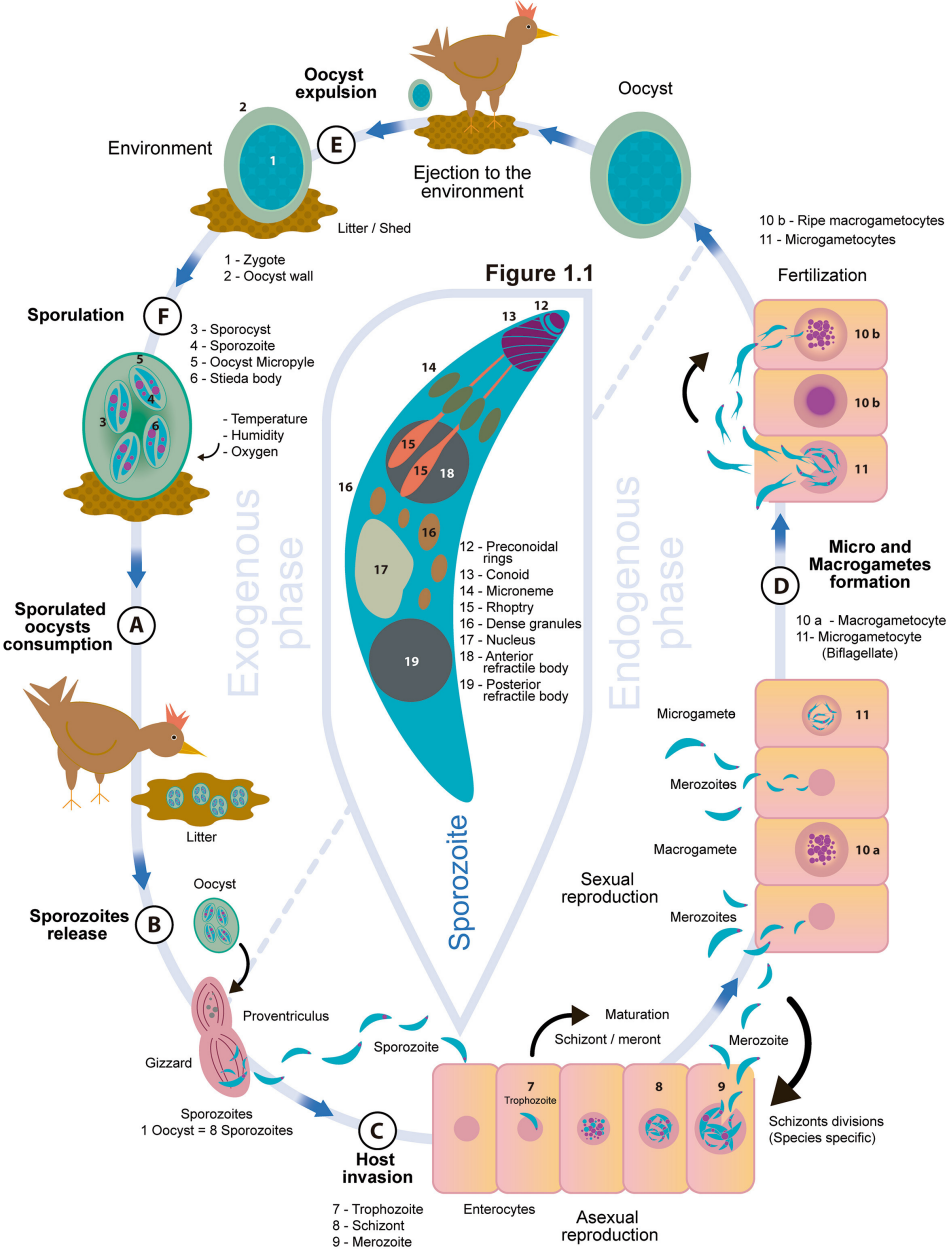
Life cycle of *Eimeria* spp and apical complex of *Eimeria*.

## Life Cycle of *Eimeria* spp.

The protozoans of the genus *Eimeria* have a direct life cycle, characterized by high tissue and host specificity, involving stages of asexual and sexual multiplication, with three development phases: the formation of schizogony (agamogony/merogony), gametogony (gamete formation for sexual reproduction), and sporogony (6).

As shown in Figure 1A, transmission occurs *via* fecal-oral route and infection begins with the ingestion of sporulated oocysts containing eight sporozoites, starting the stage called schizogony (18). The enzymatic microenvironment of the digestive tract and the mechanical action of the gizzard alter the structure and permeability of the oocyst wall (23). The sporozoites contained within each sporocyst begin to remove the protein and carbohydrate plug called the Stieda body, located in the sharp and narrow end of the sporocyst, thus allowing the sporozoites to exit into the oocyst cavity. This process, called excystation, releases them into the intestinal lumen through the oocyst micropyle (Figure 1B) (24).

The sporozoites invade the enterocytes, changing into trophozoites and starting a parasitic feeding period that lasts ~12–48 h (25, 26). The parasitophorous vacuole is formed, the trophozoite begins to enlarge, and the parasite nucleus performs multiple asexual divisions (27), forming the schizont or meront, which is full of merozoites. Approximately 3 days post infection, the mature schizont ruptures and releases the merozoites (Figure 1C) (20), which are fusiform and have an apical complex (Figure 1.1) that allows them to move and infect intestinal epithelial cells to form additional schizont generations that reproduce asexually. The number of phases of asexual reproduction is characteristic of each *Eimeria* species (Table 1) and is thought to be genetically programmed (34). The main purpose of this phase is to boost the number of merozoites within the host as preparation for the sexual reproduction phase, which is an important characteristic of every apicomplexan life cycle (35). When the asexual reproduction phase is complete, the sexual reproduction stage or gametogony begins, occurring in three events. The first is gametocytogenesis, in which gametocytes are produced from merozoites. Second, during gametogenesis, haploid micro and macrogametes are differentiated from the gametocytes. Finally, macrogametocytes are fertilized by microgametocytes (Figure 1D), producing diploid zygotes, at which point sexual reproduction is completed; meiosis proceeds, inside the protective oocyst wall, followed by mitosis to produce the infectious sporozoites (24, 35, 36).

**Table 1:** *Eimeria* species that affect poultry (*Gallus gallus)* and their main characteristics.

**Species**	**Development site**	**Pathogenicity**	**Schizogony number^¥^**	**Lesion scoring**	**Reference**
*E. praecox*	Duodenum, Jejunum	+	2	Intestinal water content, mucus and molten mucous material.	(24)^¥^, (28)
*E. acervulina*	Duodenum, Jejunum	++	4	Limited enteritis, causing loss of fluids. Poor absorption of nutrients.	(8), (29)^¥^
*E. mitis*	Ileum	+	4	Limited enteritis, causing loss of fluids. Poor absorption of nutrients.	(8), (30)^¥^
*E. maxima*	Jejunum, Ileum	++	2 –more 3	Swelling of the intestinal wall with hemorrhagic points, detachment of the epithelium.	(8), (24)^¥^, (31)^¥^
*E. brunetti*	Cecum and Rectum	+++	3	Swelling of the intestinal wall with hemorrhagic points, detachment of the epithelium.	(8), (24)^¥^
*E. tenella*	Cecum	+++	3	Thickening of the walls and blood content in the proximal end. Relaxation of the cecum. Destruction of villi, causing large hemorrhages and death. Intestine may be bloated.	(8), (24)^¥^, (32)^¥^, (33)
*E. necatrix*	Jejunum, Ileum, Cecum	+++	3	Thickening of the mucosa and intestinal lumen filled with liquid, blood and the remains of tissue. Lesions in dead birds are observable as white and black sheets (salt and pepper appearance).	(8), (32)^¥^

+ *low pathogenicity;*

++ *moderate pathogenicity;*

+++ *high pathogenicity.*

¥ *Reference for Schizogony number. Modified from Quiroz and Dantán (2)*.

The micro and macrogametocytes are morphologically different (35). The macrogametocyte grows quickly and forms a single macrogamete (37), with polysaccharide granules and lipid droplets (35); while the microgametocyte matures, breaks up and releases many small biflagellate microgametes that are a vehicle to deliver DNA (35). The amount of microgametes varies depending on the species, for example, *E. acervulina* can produce between 20 and 30, and *E. maxima* 100 or more (38). After fertilization, the oocyst is formed with an undifferentiated cytoplasmic mass which corresponds to the zygote; this mass is protected by a double wall of proteins and fats that give it great resistance to mechanical and chemical damage from the environment (2). The duration of the parasite's endogenous or internal phase is determined by the time needed to complete asexual and sexual reproduction and form oocysts.

Once the oocyst is excreted from the animal in the feces (Figure 1E), the third phase of the cycle, sporulation, takes places (39). If environmental conditions are adequate, the diploid oocyst initiates sporogony formation, which occurs in three stages (24): (1) Division of the zygote nucleus, and preparation and reorganization of the cytoplasm. This division is performed twice, giving rise to four nuclei. (2) Formation of four sporoblasts and their cytoplasmic reorganization, going through the pyramidal stage and the formation of oval sporoblasts, which will give rise to four sporocysts in total. There is no nuclear division in this stage. (3) Sporozoite formation. A single nuclear division occurs in each sporocyst and the cytoplasm is divided into two longitudinal parts to form two sporozoites inside each sporocyst with a Stieda body at its end (14) (Figure 1F). For this process to occur, optimal conditions of oxygen, temperature and humidity are required (40). Oxygen is necessary for the oocyst's respiration, as it cannot develop in anaerobic conditions. Temperature is another key factor, as oocysts have demonstrated sensitivity to high or very low temperatures (24). Studies evaluating the efficiency of sporulation at different temperatures found sporulation rates of 88.91, 88.03, and 82.44% at 25, 20, and 30°C, respectively (39). The last necessary factor is humidity, for example, a relative humidity in the environment of 75% is optimal for sporulation (40), but a dry environment causes water loss, dehydration and deformation of the oocyst wall. As a result, the zygote is pressed by the collapsed walls and there can be no normal formation of sporogony (24). Awais et al. (41) reported that in Faisalabad, Punjab, Pakistan, the incidence of coccidiosis was higher in the fall (60.02 ± 4.38) compared to other seasons, likely a result of more favorable environmental conditions that promote sporulation and survival of the oocyst; but the moisture content of the litter can also influence these rates. For example, in the case of *E. maxima*, the sporulation rate is most efficient under the driest conditions (16% moisture content), and poorest in the presence of higher moisture content (62%) (40). Sporulation time can also be influenced by the *Eimeria* species in question. Venkateswara et al. (42) evaluated the sporulation dynamics of six *Eimeria* species subjected to a temperature range between 32 and 39°C and a relative humidity of 65% to 75%. Table 2 shows the results obtained by Ventakeswara et al. (42) and others that studied sporulation time in *Eimeria* species of chickens.

**Table 2:** Comparative sporulation time (h) of *Eimeria* spp.

**Species**	**Temp. 20°C (43)**	**Temp. 29°C (43)**	**Temp. 29°C (44)**	**Temp. 32–39°C (42)**
*E. acervulina*	27	17	11.4	168
*E. mitis*	48	18	–	192
*E. maxima*	48	30	38.1	216
*E. necatrix*	48	18	19.7	96
*E. tenella*	48	18	21.2	96
*E. brunetti*	24-48	18	38.3	120

*Modified from Venkateswara et al. (42)*.

## *Eimeria* Species that Infect *Gallus gallus*

Among chickens, seven species of *Eimeria* have been described that infect different sections of the intestine (Table 1) (6, 7). Each species of the parasite has a preference for a specific site in the gastrointestinal tract (45), as well as differentiating characteristics in the appearance of macroscopic lesions, the morphology of the oocysts, the minimum sporulation time, the minimum prepatent period (time between the bird's infection with a sporulated oocyst and the shedding of the first oocysts into the environment through feces) (46), the size of the schizont and the location of the development of the parasite in the intestinal epithelium (39, 47). Within the described species, there are three particularly relevant species in broilers: *E. acervulina*, which develops in the epithelial cells in the proximal region of the small intestine, mainly in the duodenum (48), *E. maxima*, which targets the intermediate region of the intestine and is easily recognizable due to the size of its oocysts (the largest), and *E. tenella*, which infects the cecum and causes bloody diarrhea (49).

While the majority of research has focused on the seven species mentioned above, in recent years, three cryptic *Eimeria* genotypes have been identified, initially in Australian chicken populations, and have now been proposed as novel species. They were initially characterized as novel operational taxonomic units (OTUs) (50). Following their discovery, these OTUs were divided into three distinct phylogenetic clusters, denoted by the abbreviations OTUx, y, and z. (51). The differences in biological traits, genetic and antigenic diversity were evaluated, comparing these directly with the seven recognized *Eimeria* spp. Significant differences were found, enough to propose them as new *Eimeria* species that can infect *Gallus gallus*, called *Eimeria lata* n sp. (previously OTUx), *Eimeria nagambie* n sp. (previously OTUy), and *Eimeria zaria* n sp. (previously OTUz) (52).

## Pathology and Diagnosis of Avian Coccidiosis

It has been shown that the degree of infection and the clinical signs of coccidiosis are influenced by multiple factors including the species of *Eimeria*, the infective dose, host-parasite interactions and environmental conditions of the poultry barn.

### The *Eimeria* Species

The pathogenicity of different *Eimeria* ranges from moderate to severe (36), some species may cause loss of fluids and a decrease in nutrient absorption (*E. acervulina* and *E. mitis)*, swelling of the intestinal wall with petechiae and loosening of the epithelium (*E. brunetti* and *E. maxima)* or complete destruction of villi, producing hemorrhages and death (*E. necatrix and E. tenella)*, each species causes recognizable and distinct signs of coccidiosis, independent of the other species (8, 53).

### Infective Dose

Coccidial infections are self-limiting and depend largely on the number of sporulated oocysts ingested (6). Several studies have shown that there is an optimal dose, such that the reproductive potential of the parasite is met and they efficiently replicate within the epithelial cells. Very high doses of ingestion can cause the so-called “crowding effect,” interrupting the continuity of the parasite's life cycle, while still generating intestinal damage (54–56). For example, Williams in 2001 (55) was able to characterize the reproductive potential of each species of *Eimeria* under experimental conditions, using infective doses of 903, 16, 39, 14, 16, 16, or 72 sporulated oocysts, of *E. acervulina, E. brunetti, E. maxima, E. mitis, E. necatrix, E. praecox* or *E. tenella*, respectively.

### Host

The host is a pivotal factor of *Eimeria* infections in poultry (6). Some *Eimerian* parasites are highly immunogenic in chickens, and primary infections can stimulate protective immunity to subsequent challenge by the homologous parasite (57). Lillehoj in (58) used two chicken lines SC (B^2^B^2^) and FP (B^15^B^21^), to demonstrate that age and host genetic background can affect the outcome of coccidial infections. These two lines were subjected to an experimental infection with different doses of sporulated oocysts of *Eimeria tenella*, where is was found that in a reinfection process at different ages, the FP line was more resistant to *Eimeria* than the SC line when the primary innoculation contained a high dose of oocysts. Further, they determined that older animals demonstrated total immunity to *Eimeria*.

Additionally, some publications suggest that host sex influences the prevalence of coccidiosis; for example, Hadas et al. (59) and Wondimu et al. (60) report a prevalence of coccidiosis in farms of Gondar Town, Ethiopia that is relatively higher in male (44.3–43.6 percent) than female chicken (42.4–41.2 percent), but no significant statistical difference was found in either study.

### Poultry House Environment

There are specific factors that jeopardize and increase the spread of the parasite, including inadequate biosecurity protocols and poor hygiene of both personnel and equipment (60, 61). Sanitization plays a major role in reducing the dissemination of the parasite (62), as the most frequent mode of transmission of oocysts is through mechanical vectors such as movement of personnel or equipment between farms, and the presence of rodents and insects such as flies and beetles (6, 63).

### Diagnosis

**Figure 2:**
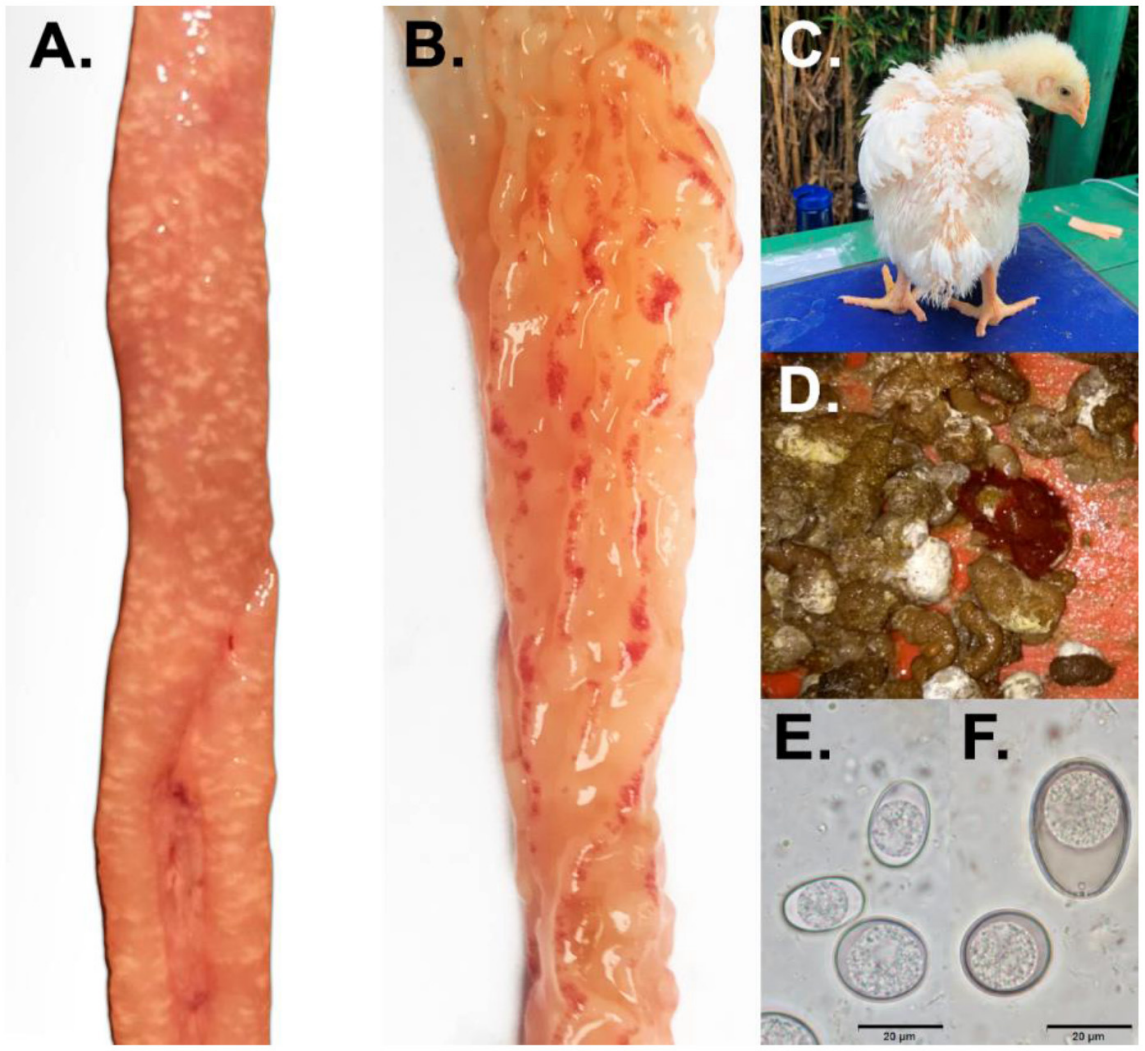
Classical methods for the evaluation of *Eimeria* infections. **(A,B)** Gross lesion *E. acervulina* and *E. tenella*. **(C)** Bird with coccidiosis. **(D)** Feces with blood. **(E,F)** Shape of oocysts of *Eimeria* spp.

Correct identification of *Eimeria* species is important for the diagnosis and control of the disease (64) and from a commercial standpoint, a diagnosis of coccidiosis is required when the gross lesions are evident (6, 49). Classical methods for the evaluation of *Eimeria* infections include macroscopic diagnosis with observation of clinical signs in infected animals, the location and appearance of gross lesions during necropsy; and microscopic diagnosis, which focuses on evaluating the size and shape of oocysts (Figure 2) (49). Sometimes the evaluation of other developmental stages in microscopic smears is also included (65). In addition, when greater diagnostic precision is needed, molecular diagnostics can be included (35, 66, 67).

### Clinical Signs

Coccidia invade the intestinal mucosa and induce a certain degree of epithelial cell damage and inflammation. Meronts, gamonts, and oocysts cause marked histological alterations of host intestinal epithelial cells over a short time period including distortion, rupture, separation from adjacent cells, and sloughing (68). Infected birds present with ruffled feathers and signs of depression or drowsiness (Figure 2). Additionally, feed and water consumption are decreased, and the feces may be watery, whitish and occasionally bloody (69). This results in dehydration, impaired weight gain, and in the absence of treatment, death (61). Additionally, there is the problem of malabsorption due to reduced brush border enzyme activity (70) and disruption of intestinal integrity (71).

Infection can cause other intestinal changes, as well; for example, an inoculation with E. acervulina and E. maxima oocysts increased the size and number of goblet cells along ileal crypts in broilers (72). Goblet cells represent an important defense mechanisms in the intestinal tract (73), secreting glycoproteins of high molecular weight called mucins (74). Mucins are the first line of defense against intestinal pathogens and act to protect the epithelium from pathogens and irritants in the intestinal lumen (75). Similarly, it has been reported that when *E. tenella* invades cecal epithelial cells, the cecum increases the rate of mucus production and promotes a protective phenotype as an immunological reaction against the parasite (76, 77). However, this increase in mucin production can also be harmful, promoting secondary colonization by other pathogens such as *Clostridium perfringens* (72, 78, 79), *Salmonella* and certain viruses like Marek's disease virus or infectious bursal disease virus (6, 80). This has the effect of further altering intestinal health by impairing metabolism and nutrient absorption (81).

For the evaluation of gross lesions, a standardized intestinal lesion scoring technique is used (82), which is based on giving a score on a scale of zero to four, with the goal of obtaining a numeric classification of the gross lesions caused by each *Eimeria* species (65, 67–83) (Figure 2). For this scoring system, the entire intestine of the bird must be evaluated, beginning with the duodenum. The mucus and serous membranes are examined to detect lesions, and a good light source (solar or lamp) is essential for reliable scoring (49). Table 3 summarizes the changes visible in the walls of infected organs by oocysts of *E. acervulina, E. maxima* and *E. tenella* and respective lesion scoring. Generally, a set number of birds per flock are assessed (between 5 and 6) and the individual scores are added for all *Eimeria* spp. (84). This is a laborious method, it can be subjective, and it needs experienced personnel to obtain an accurate outcome. However, it is still the most widely used diagnostic method (49).

**Table 3:** Description of changes and score of intestinal lesions due to infection with *Eimeria* spp.

***Eimeria*** **spp**.	**Score**	**Description**
*acervulina*	0	No gross lesions
	1	The presence of white scattered lesions (not more than five per square centimeter) oriented transversely or ladder-like, clearly visible on the mucosal surface of the duodenal loop. White lesions contain developing oocysts.
	2	Lesions are much closer together, but not coalescent; The intestinal walls show no thickening. With a good light source, these distinctive transversely elongated white plaques may be readily recognized on the serosal as well as the mucosal surface
	3	Lesions are more numerous and beginning to coalesce. The intestinal wall is thickened and the contents are watery. Lesions may extend as far posterior as Meckel's diverticulum.
	4	Lesions are coalescing in the portion of the duodenum attached to the gizzard and the mucosal wall is grayish, the intestinal wall is greatly thickened, and the intestine is filled with a creamy exudate which may bear large numbers of oocysts.
*maxima*	0	No gross lesions
	1	Small red petechiae may appear on the serosal side of the mid-intestine. There is no ballooning or thickening of the intestine, though small amounts of orange mucus may be present
	2	Serosal surface may be speckled with numerous red petechiae; intestine may be filled with orange mucus; little or no ballooning of the intestine; thickening of the wall
	3	Intestinal wall is ballooned and thickened. The mucosal surface is roughened; intestinal contents are filled with pinpoint blood clots and mucus
	4	The intestinal wall may be ballooned for most of its length; contains numerous blood clots and digested red blood cells giving a characteristic color and putrid odor; the wall is greatly thickened; dead birds are recorded with this score
*tenella*	0	No gross lesions
	1	Very few scattered petechiae on the cecal wall; no thickening of the cecal walls; normal cecal contents present.
	2	Lesions more numerous with noticeable blood in the cecal contents; cecal wall is somewhat thickened
	3	Large amounts of blood or cecal cores present; cecal walls greatly thickened; little, if any, fecal contents in the ceca
	4	Cecal wall greatly distended with blood or large caseous cores; fecal debris lacking or included in cores

*Modified from Johnson and Reid (82)*.

### Microscopic Diagnosis

Scrapings of the intestinal mucosa can be taken to evaluate the presence and shape of oocysts (65) or this count can be done using droppings (85). Typically, the intestinal lesion score is complemented with counts of oocysts per gram (OPG) of feces or poultry litter through the McMaster technique (86). It is believed that the correlation between lesion scoring and productive performance is stronger than the relationship between oocyst counts (OPG) and performance (49). Perhaps, oocyst shedding does not correlate well with decreased body weight gain or intestinal lesion scores because high doses of *Eimeria* can result in a crowding effect that reduces oocyst shedding while still causing significant damage to the intestine (55). Regardless, greater accuracy is needed to determine the level of intestinal lesions at which performance begins to be impacted, especially when subclinical conditions are present (49).

**Figure 3:**
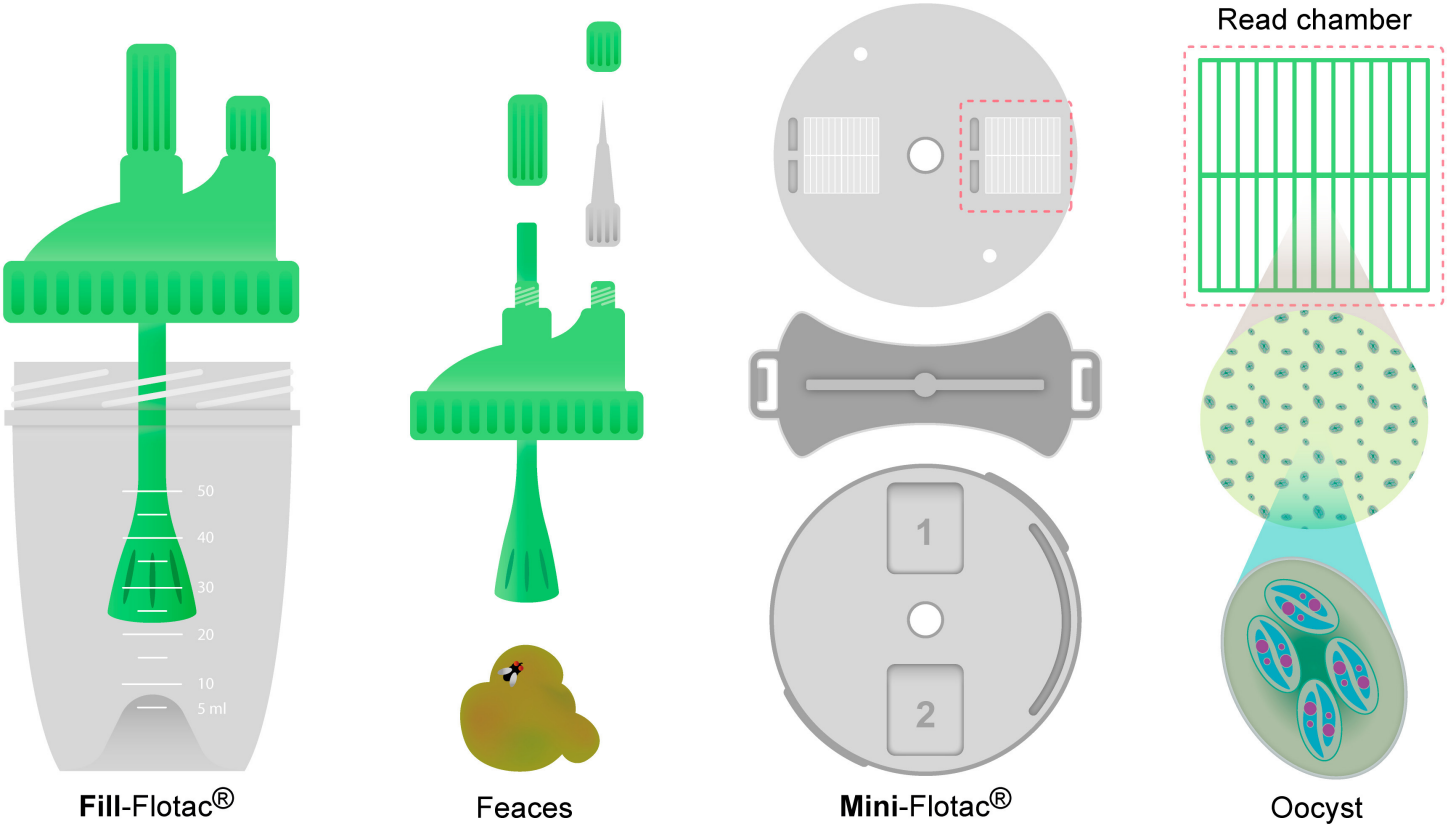
Mini-flotac^®^ diagram. Fill- flotac^®^ with lid, filter and homogenizer and Mini-flotac^®^ with disc, two reading chambers and key.

Recently, the Mini-FLOTAC (Figure 3) was developed as a new method for qualitative and quantitative diagnosis of infections by helminths and protozoans in several mammal hosts. This is a useful technique to process large amounts of samples rapidly at the laboratory or at the farm (87). The Mini-FLOTAC technique is based on flotation principles with saturated solutions, using a device with two components, the Fill-FLOTAC and reading chamber (86). The Fill-FLOTAC is a clear plastic container with a capacity of 70 mL, used to carry out the first four steps of the technique i.e., sample collection and weighing; homogenization; filtration; and filling of the chambers. The other component is the Mini-FLOTAC reading chamber, which has two components (the base and the disk with two reading chambers, 1 mL each, with ruled grids on the surface, which divide each chamber into 12 sections) and two accessories (the key and the microscope adaptor) (87).

The microscope reading is done at a magnification of 100x (86), up to 400x when there are small sized protozoa (87). This technique has become an alternative to the McMaster method, especially in cases where greater accuracy is needed (60), and has been used successfully in different species including goats and horses (88, 89). According to Bortoluzzi et al. (86), who compared the precision of the McMaster technique and the Mini-FLOTAC to quantify *Eimeria maxima* oocysts, the Mini-FLOTAC is a reliable and precise method of quantification for this species with the detection limit ranging between 100 and 500 oocysts per gram of excreta (OPG). Other works like Das et al. (90), found the Macmaster technique to be less sensitive than Mini-FLOTAC when the load was 50 OPG, whereas there was no difference observed when 100, 250, 300, 450, 625 and 1,250 OPG were present. Additionally, this study concludes that the McMaster method is faster than Mini-FLOTAC and both McMaster and Mini-FLOTAC underestimate true OPG counts.

### Molecular Diagnosis

In practical conditions, *Eimeria* infections are often caused by more than one species, with similar pathological characteristics, making diagnosis in the field difficult (64). This means that methodologies that can be more sensitive and less subjective are very important for correct diagnosis (91). Molecular biology techniques offer improved diagnostic precision in many cases. One example is polymerase chain reaction (PCR) based identification of *Eimeria*; the amplification of regions of the internal transcribed space 1 (ITS1) of the ribosomal DNA (92–94) is now a widely available technique. The ITS is a piece of non-functional RNA located between structural ribosomal RNAs (rRNA) on a common precursor transcript. This region of the genome contains several segments used for *Eimeria* spp. identification, including the 5' external transcribed sequence (5'ETS), 18S rRNA, ITS1, 5.8S rRNA, ITS2, 26S rRNA, and finally the 3'ETS. Because it is easy apply this technique with only small amounts of DNA and because there is a high degree of variation between closely related species, the ITS region is widely used in molecular phylogeny and taxonomy (91). Other molecular techniques that have been reported for the identification of *Eimeria* species have been Random Amplified Polymorphic DNA (RAPD) (95), Sequence Characterized Amplified Region (SCAR) markers (96, 97), quantitative PCR (7) and Loop-Mediated Isothermal Amplification (LAMP) (97, 98). Furthermore, multiplex PCR techniques for detecting the seven *Eimeria* species of interest have been described, which combine primers for each species in a single reaction (99). Given that this is a parasite with a wide epidemiological distribution and with reports of taxonomic variants throughout the world (100), continued study is necessary. Continued advances in Next Generation Sequencing techniques (NGS) are allowing for precise identification of emerging or region-specific *Eimeria* spp. as well as facilitating other avenues of research for control of coccidiosis (66, 67).

## Methods Used to Control Avian Coccidiosis

The prevention and control of coccidiosis is based on the use of vaccines, natural feed additives, prophylactic anticoccidial drugs, and improved handling practices on farms. Some beneficial practices include cleaning and disinfection of facilities, and adequate ventilation and clean water, all of which contribute to maintaining litter conditions that minimize the sporulation of oocysts (10). Prevention (prophylaxis) has traditionally been a pillar of broiler chicken production (101), relying on anticoccidials to avoid outbreaks of the disease (62).

### Control With Anticoccidial Agents

Since the 1950s, it has been common to raise broiler chickens and turkeys with feed anticoccidials. According to Agri Stats Inc. (Fort Wayne, IN), in the late 1990s, 99% of broiler chickens were raised with an anticoccidial drug in one or more phases, and this practice is still prevailing in many markets (102). However, trends in some markets are changing and today, some of the largest producers of broiler chickens in the world, like the USA, are raising up to 60% of broilers without anticoccidials (103).

Anticoccidials, based on their mode of action, may be divided into coccidiostats and coccidicides. Coccidiostats halt the development of the parasite, compromising its replication and growth, but their effect can be reversible, as removal from the diet can lead to the re-emergence of the disease. Coccidicides are characterized by killing or causing irreversible damage to the parasite (62).

Anticoccidials may also be classified into two categories according to their origin (10, 104): (1) synthetic compounds, which are produced by chemical synthesis and have a specific mode of action against the metabolism of the parasite (10); (2) polyether or ionophore antibiotics, which are produced by the fermentation of *Streptomyces* spp. or *Actinomadura* spp., which generally destroy coccidia by interfering with the passage of monovalent or divalent ions including sodium, potassium, calcium and magnesium, through the parasite's cell membrane (81, 102, 105). Additionally, in the market there are “mixed products” that are comprised of a combination of the two (10). Table 4 provides further characterization of these categories.

**Table 4:** Anticoccidial agent's classification.

	**Category**	**Anticoccidial agent**	**Recommended dose (ppm)-Broiler**
Ionophores	Monovalent	Monensin	100–120
		Narasin	60–80
		Salinomycin	44–66
	Monocyclic glycosidic	Maduramicin	5–6
		Semduramicin	25
	Divalent	Lasalocid	75–125
Chemicals		Amprolium	125–250
		Aprinocid	60
		Clopidol	125
		Decoquinate	30
		Diclazuril	1
		Dinitolmide (zoalene)	125
		Halofuginone	3
		Nequinate (methyl benzoquate)	20
		Nicarbazin	125
		Robenidine	33
Mixed	Synthetic with ionophore	Salinomycin/nicarbazin^*^	50
		Narasin/nicarbazin^†^	54–90 (combination)
		Maduramicin/nicarbazin	3.75–40
		Semduramicin/nicarbazin^*^	15–40
		Monensin/nicarbazin^*^	40
	Synthetic with synthetic	Meticlorpindol/methylbenzoquate^*^	110

*Adapted from Peek and Landman (10);*

* *Recommended dose commercial product (106–110).*

† *Feed additive compendium (111)*.

The extensive use of these anticoccidials prophylactically has resulted in a loss of efficacy of these compounds, triggered by increasing resistance by the parasite (11). To overcome this, anticoccidials are now used under programs called dual (or shuttle) or straight rotation. In the first program, two or more anticoccidials, usually with different modes of action, are alternated in the different feeds supplied during the chicken life cycle; whereas in the second program, the same drug is used continuously throughout a production cycle, but is changed for an alternative drug after one or several flocks (2, 8, 10).

### Control of Coccidiosis in Chickens by Vaccination

Infection with *Eimeria* spp. triggers a variety of mechanisms in a host's immune system, resulting in an effective, long-lasting, but species-specific immunity (68). Generally, generating an immune response against *Eimeria* requires a large number of inoculating oocysts, with the exception of *E. maxima*, which is considered to be highly immunogenic and requires only a small number of oocysts to induce strong immunity (68). Additionally, the early endogenous stages of the parasite life cycle are considered to be more immunogenic than the later sexual stages (112). The immune system of birds is well-developed and when facing a challenge from an intestinal parasite like *Eimeria* (68), the gut-associated lymphoid tissue (GALT) is the first line of defense. This system has evolved into a specialized immune com-plex with organs such as Peyer's patches (PP), the bursa of Fabricius, and the cecal tonsils, hosting a variety of specialized immune cells such as epithelial, NK, and den-dritic cells (9). Infection stimulates a host response that begins with a non-specific immune response, mediated by macrophages, granulocytes, natural killer (NK) cells and soluble factors such as serum proteins. This precedes the development of an antigen-specific memory immune response mediated by lymphocytes (113) and their secretions, in the form of antibodies and cytokines (9, 68). The works of Rose et al. (114, 115) and Lillehoj and Choi (116) demonstrate the progression of the innate and adaptive responses to *Eimeria* infection in mouse and chicken models.

The first study that showed resistance in chickens against infection with *E. tenella* was reported by Beach and Corl in 1925; but it was not until 27 years later that the first live commercial vaccine against coccidiosis (CocciVac®) was launched in the United States (17, 62, 81, 105). Vaccination is included in coccidia control programs with the aim of inducing an immune response, generating protection against subsequent challenges with *Eimeria* spp., and decreasing the severity of coccidiosis (9, 10, 83).

Different vaccines are available on the market, including live virulent, live attenuated, and non-infectious derivatives of the parasite (subunit vaccines) (9, 117). Live vaccines use the oral introduction of low doses of *Eimeria* oocysts to stimulate humoral and cellular responses from the host immune system. The ingested oocysts generate a variety of antigens in different stages of the *Eimeria* life cycle to stimulate this response (8), with the cellular response being the most important in terms of resistance to the disease. This is supported by a scientific paper published by Lillehoj (118) in which birds were treated with Cyclosporin A to suppress cell-mediated immune responses, or treated with hormones to abrogate their humoral response by interfering with the bursa. The result was that birds with a hormonal bursectomy showed no alteration in their response to challenge with *Eimeria tenella*, while birds who had been previously treated with cyclosporin a showed increased susceptibility to the parasite.

These and other studies are crucial in the development of future vaccines. These studies are important to more fully understand the nature of the immune response against the parasite, as well as other interactions between the host and parasite (17, 62–65, 67–119).

### Virulent Vaccines

Virulent strains isolated from the field without any alteration in their pathogenicity can be used as vaccines, as well (62), being highly effective and relatively cheap to produce. However, this practice also risks compromising flock performance and occurrence of clinical disease if managed incorrectly (120, 121). In mass vaccination, it is important to carefully standardize the dosage methods and conditions, enabling uniform inoculation within the flock, increasing oocyst shedding into the litter and reinfection of the flock. Animals that do not receive an appropriate dose of the vaccine may perpetuate subsequent infections that can cause asynchronous immunity, compromising the performance of the birds and increasing future susceptibility to the disease (9). These drawbacks have limited the use of virulent vaccine strains and they are not currently licensed for use in the European Union (122). However, this type the vaccine is used widely across much of North America, as well as in parts of Africa and Asia (121, 123).

### Live Attenuated Vaccines

The goal of attenuation is to decrease the pathogenicity of the parasite and therefore, its deleterious effects on the host. Several methods of attenuation have been used, including, selection for precociousness (124), irradiation (125), chemical treatment (126) and serial passage in chicken embryos (127). Precocious lines of Eimeria are characterized by a shortened endogenous life cycle due to the elimination of one or more schizogonies, leading to less damage to the intestinal tract and decreased production of oocysts. By selecting the first oocysts that are excreted in the feces to inoculate chickens during the second pass and repeating this process over and over, the reproductive potential and the pre-patent period of the Eimeria species selected is lowered. Along with these reductions there is a concomitant reduction in pathogenicity while the immunogenicity is retained (124). This type of vaccine is used extensively in much of Europe, as well as parts of Africa, Asia and Australasia, primarily with layer and breeder stock owing to their relative cost and limited productive capacity (123).

### Subunit Vaccine

Identification of protective antigens is essential for the development of new vaccines against coccidiosis (128). Isolated and purified epitopes from virulent strains have been used in anticoccidial vaccines, mainly native or recombinant proteins expressed during various stages of development (sporogonies, merogonies, gametogonies) of the *Eimeria* parasite (62). This is particularly useful for maternal immunization, stimulating the production of large amounts of immunoglobulin Y (IgY), which is then transferred through the yolk of the egg, providing protective immunity to its offspring. This vaccination strategy can decrease the excretion of oocysts in birds challenged with *Eimeria maxima* up to 83%, and can provide some cross-protection against heterologous species like *E. tenella* and *E. acervulina* (128).

Attempts to develop next-generation recombinant anticoccidial vaccines have led to the identification of many potential vaccine antigens. Small-scale vaccination trials using antigens in recombinant protein, DNA or live-vectored formulations have been reported to achieve 30–90% reductions in parasite replication and/or gut lesion score, or comparable improvements in feed conversion ratio and/or body weight gain (122). However, one major constraint in deployment of such antigen-specific vaccines is an appropriate and effective delivery system (51). Several possible vectors for oral administration, including *Bacillus, Salmonella*, transgenic *Eimeria* and yeasts such as *Saccharomyces cerevisiae*, are currently in development and could be appropriate (51, 129, 130).

Currently, vaccination is a practice that is being promoted mainly due to demand for products satisfying a “No antibiotics, ever” label (51). In the past, it was a common practice only in breeder pullets and turkeys (131); this trend has been changing because public and legislative pressures are encouraging the search for cost-effective alternatives to anticoccidial drugs in broiler production, especially in countries such as the US, where (unlike the EU) ionophores are regulated as antibiotics (123). In response to these external pressures, 35–40% of US broiler companies have adopted annual cycles where two out of every six flocks receive anticoccidial vaccination instead of drugs (104). This practice is known as a bio-shuttle program in which vaccination of broilers on day of hatch is followed by the administration of grower and finisher diets containing anticoccidial drugs (132). This allows producers greater control in managing the risk of outbreak posed by the use of non-attenuated vaccines.

Another important advantage to using live vaccines for the control of coccidiosis is the replacement of *Eimeria* populations residing in the poultry house. This often has the effect of restoring the susceptibility of the *Eimeria* population in the house to traditional anticoccidial drugs, as evidenced in the work of Chapman and Jeffers (12). This study was an anticoccidial resistance trial in broiler chickens, tracking five consecutive flocks on a rotation schedule of anticoccidials. The use of vaccination in conjunction with salinomycin (ionophore) and diclazuril (chemical), restored sensitivity to these anticoccidials: they demonstrated that the anticoccidial programs that followed the vaccination program had improved sensitivity of the parasite to the anticoccidials.

### Natural Products for Coccidiosis Control

Currently, difficulties including resistance to the cost of anticoccidials (133); consumer pressure for poultry products labeled as “antibiotic-free,” “no antibiotics ever,” or “raised without antibiotics”; (134), as well as the pathogenicity of live vaccines, is leading poultry producers worldwide to intensify their search for strategies that include safe, effective and economically viable alternatives for controlling coccidiosis (11, 133).

These alternatives include prebiotics, probiotics, essential oils, organic acids, antioxidants and nanobiotics (plant nanoparticles that have been used as antibacterial agents) (81, 135). Many of these compounds are used as dietary supplements with various applications including immune system stimulation, and anti-inflammatory and antioxidant action (2, 133). For example, the work Ali et al. (69), showed the anticoccidial effect of garlic (*Allium sativum*) and ginger (*Zingiber officinale*) against experimentally induced coccidiosis. Feed intake, body weight and feed conversion ratio (FCR) were significantly improved in ginger and garlic supplemented birds compared to the positive control (infected without additives). Similarly, oocyst shedding, lesion score and histopathology of the small intestines improved in ginger and garlic supplemented birds after challenge.

Table 5 shows some natural compounds that have been used for *Eimeria* control, their mechanism of action, and their efficacy in controlling coccidiosis (140). Mixed results in many studies show the need for further research into the potential of these alternative control strategies. For example, Scheurer et al. (137) examined three phytogenic compounds (oregano; combination of *Curcuma*, saponins, and inulin; Quillaja), and showed that there were no effects against Coccidiosis. Similarly, Idris et al. (141) reported that the use of essential oils as an alternative to anticoccidials is limited because of their antinutritional factors, toxicity, low dose effectiveness and their reduced protective response.

**Table 5:** Alternative products with potential anticoccidial effect.

**Additive**	**Major component(s)**	**Doses/concentration**	**Action mode**	**Effect**	**Type of study**	**Reference**
Artemisia (essential oil)	β-thujone: 64%; 1-8 cineol: 18%; p-cymene: 9.6%; sabinene: 7.8%	0.3–20 mg/ml	Induction of oxidative stress	Reduces the number of oocysts	*In vitro*	(136)
Clove (essential oil)	Eugenol: 72.9%; eugenyl acetate: 5.8%	0.3–20 mg/ml	Unknown	Reduces the number of oocysts	*In vitro*	(136)
Turmeric combined with saponins and inulin	*Curcuma longa, Quillaja saponaria, Cichorium intybus*	1,000 (ppm) in feed	Stimulation of the system by inactivation of reactive nitrogenous radicals	Has no significant effect on lesion scoring	*In vivo* Broiler Research facility	(137)
Oregano (essential oil)	*Oreganum vulgare*	200 (ppm) in feed	Mucosal immunity stimulation	Has no significant effect on lesion scoring	*In vivo* Broiler Research facility	(137)
Quillajacea (plant extract)	*Quillaja saponaria*	1,000 (ppm) in feed	Antiprotozoal activity (It binds to the protein of the membrane of protozoal cells)	Has no significant effect on lesion scoring	*In vivo* Broiler Research facility	(137)
S-nitrosoglutathione (GSNO)	–	20 mM	Inhibits the sporulation process of *E. tenella* oocysts	Interrupts the sporulation process for 10 h after the initial sporulation; no effect after 12 h	*In vitro*	(138)
Lespedeza cuneata (plant extract)	Condensed tannins	1–2 and 4% diet supplement	Tannins have anticoccidial activity against the parasite	No significant difference in the number of oocysts	*In vivo* Broiler Research facility	(139)
Tea tree (essential oil)	Terpinen-4-ol: 40%; gamma-terpinen: 21.4%	0.3–20 mg/ml	Unknown	Reduces the number of oocysts	*In vitro*	(136)
Thyme (essential oil)	Thymol: 36.6%; p-cimène: 16.5%	0.3–20 mg/ml	Unknown	Reduces the number of oocysts	*In vitro*	(136)

*Modified from Kadykalo et al. (140)*.

Further investigations should explore their mechanisms of action, and their protective response should be evaluated alone or in combination with a vaccine (141), as future control strategies are likely to include combinations of products as replacements for traditional anticoccidials.

## Conclusion and Perspectives

Coccidia is considered to be a ubiquitous parasite in poultry production, as reflected by the prevalence and frequency of *Eimeria* infection in various regions of the world. For example, Colombia reports a frequency of 92.8% (142); 90% in Argentina (143); 92% in Romania (47), 79.4% in North India (144), 65.8% in East China (145), and 78.7% in South Korea (146). Even in countries with lower reported incidence, *Eimeria* is a frequent and expensive problem (59, 60, 147, 148). This review have shown how the control in poultry was achieved successfully, by a combination of improved management, the prophylactic use of drugs, and vaccination. However, because the parasite has not been totally eradicated from commercial facilities where animals are reared and is still capable of causing performance and health issues due to the generation of resistance to anticoccidials through the rotation programs. Moreover, information gleaned from molecular assays can guide the poultry producers in managing disease by allowing informed decisions on which anticoccidial compounds (traditional or naturals) or live oocyst vaccines should be used in the field. For this reason, is important to update and expand the understanding of basic concepts about *Eimeria*, which causes a negative impact on poultry farming globally. Further, continued basic and applied research based on molecular methodologies and field test, that support the identification and characterization of different *Eimerias* in order to achieve more accurate identification of the different *Eimerias*. In this way, alternative control strategies focused on global trends in production without antibiotics needs to be developed.

## Author Contributions

JC-G and LG-O: conceptualization and article structuring. CM-P, JN-R, and SL-O: writing and topic review. CM-P and JN-R: design and creation of figures. All authors listed have made a substantial, direct, and intellectual contribution to the work and approved it for publication.

## Conflict of Interest

LG-O was employed by the company Alura Animal Health and Nutrition and CM-P was employed by the company Solla S.A. The remaining authors declare that the research was conducted in the absence of any commercial or financial relationships that could be construed as a potential conflict of interest.

## Publisher's Note

All claims expressed in this article are solely those of the authors and do not necessarily represent those of their affiliated organizations, or those of the publisher, the editors and the reviewers. Any product that may be evaluated in this article, or claim that may be made by its manufacturer, is not guaranteed or endorsed by the publisher.

## References

[1] Bogosavljevic-Boskovic S Mitrovic S Djokovic R Doskovic V Djermanovic V. Chemical composition of chicken meat produced in extensive indoor and free range rearing systems. Afr J Biotechnol. (2010) 9:9069–75. 10.5897/AJB10.1084

[2] Quiroz-Castañeda RE Dantán-González E. Control of avian coccidiosis: future and present natural alternatives. BioMed Res Int. (2015) 2015:430610. 10.1155/2015/430610 25785269 PMC4346696

[3] USDA. Livestock and Poultry: World Markets and Trade. United States Department of Agriculture Foreign Agricultural Service January 10, 2020. (2020). Available at: https://apps.fas.usda.gov/psdonline/circulars/livestock_poultry.pdf. (accessed: March 10, 2021).

[4] O'neill BC Dalton M Fuchs R Jiang L Pachauri S Zigova K. Global demographic trends and future carbon emissions. Proc. Natl. Acad. Sci. (2010) 107:17521–26. 10.1073/pnas.1004581107 20937861 PMC2955139

[5] Godfray HCJ Beddington JR Crute IR Haddad L Lawrence D Muir JF. Food security: the challenge of feeding 9 billion people. Science. (2010) 327:812–18. 10.1126/science.1185383 20110467

[6] Cervantes HM McDougald L.R Jenkins MC Coccidiosis, In: Diseases of Poultry, Volume II. Fourteenth Edition. Editor-in-chief David E. Swayne: John Wiley & Sons, Inc. (2020). p. 1193–217.

[7] Vrba V Blake DP Poplstein M. Quantitative real-time PCR assays for detection and quantification of all seven *Eimeria* species that infect the chicken. Vet Parasitol. (2010) 174:183–90. 10.1016/j.vetpar.2010.09.006 20888693

[8] Chapman HD. Milestones in avian coccidiosis research: a review. Poult Sci J. (2014) 93:501–11. 10.3382/ps.2013-03634 24604841

[9] Shivaramaiah C Barta JR Hernandez-Velasco X Téllez G Hargis BM. Coccidiosis: recent advancements in the immunobiology of *Eimeria* species, preventive measures, and the importance of vaccination as a control tool against these Apicomplexan parasites. Vet Med Res Rep. (2014) 5:23–34. 10.2147/VMRR.S57839 32670843 PMC7337151

[10] Peek HW Landman WJM. Coccidiosis in poultry: anticoccidial products, vaccines and other prevention strategies. Vet Q. (2011) 31:143–61. 10.1080/01652176.2011.605247 22029884

[11] Abbas RZ Iqbal Z Blake D Khan MN Saleemi MK. Anticoccidial drug resistance in fowl coccidia: the state of play revisited. World Poult Sci J. (2011) 67:337–50. 10.1017/S004393391100033X30886898

[12] Chapman HD Jeffers TK. Restoration of sensitivity to salinomycin in *Eimeria* following 5 flocks of broiler chickens reared in floor-pens using drug programs and vaccination to control coccidiosis. Poult Sci J. (2015) 94:943–46. 10.3382/ps/pev077 25796273

[13] Williams RB. A compartmentalised model for the estimation of the cost of coccidiosis to the world's chicken production industry. Int J Parasitol. (1999) 29: 1209–29. 10.1016/S0020-7519(99)00086-7 10576573

[14] Müller J Hemphill A. In vitro culture systems for the study of apicomplexan parasites in farm animals. Int J Parasitol. (2013) 43:115–24. 10.1016/j.ijpara.2012.08.004 23000674

[15] Suarez CE Bishop RP Alzan HF Poole WA Cooke BM. Advances in the application of genetic manipulation methods to apicomplexan parasites. Int J Parasitol. (2017) 47:701–10. 10.1016/j.ijpara.2017.08.002 28893636

[16] Levine ND. Protozoan Parasites of Domestic Animals and Man. Minneapolis: Burgess Publishing Company. (1961).

[17] Williams RB. Review Article: anticoccidial vaccines for broiler chickens: pathways to success. Avian Pathol. (2002) 31:317–53. 10.1080/03079450220148988 12396335

[18] Haug A Gjevre AG Skjerve E Kaldhusdal M. A survey of the economic impact of subclinical *Eimeria* infections in broiler chickens in Norway. Avian Pathol. (2008) 37:333–41. 10.1080/03079450802050705 18568662

[19] Khazandi M. Title The Eimeria-Host Cell Interaction in Broiler Chickens. Roseworthy, SA: University of Adelaide. (2006).

[20] Nabian S Arabkhazaeli F Seifouri P Farahani A. Morphometric analysis of the intestine in experimental coccidiosis in broilers treated with anticoccidial drugs. Iran. J. Parasitol. (2018) 13:493–99. 30483343 PMC6243159

[21] Madlala T Okpeku M Adeleke MA. Understanding the interactions between Eimeria infection and gut microbiota, towards the control of chicken coccidiosis: a review. Parasite. (2021) 28:1–10. 10.1051/parasite/2021047 34076575 PMC8171251

[22] Bowman D. Georgis'Parasitology for Veterinarians. St Louis, MO: Elsevier. (2014).

[23] Chapman HD. Studies on the excystation of different species of eimeria in vitro. Zeitschrift Parasitenkd Parasitol Res. (1978) 56:115–21. 10.1007/BF00930742 695823

[24] Kheysin YM. Life Cycles of Coccidia of Domestic Animals. New York, NY: Elsevier. (1972).

[25] Rose ME Lawn AM Millard BJ. The effect of immunity on the early events in the life-cycle of Eimeria tenella in the caecal mucosa of the chicken. Parasitology. (1984) 88:199–210. 10.1017/S0031182000054470 6718052

[26] Trout JM Lillehoj HS. Eimeria acervulina infection: evidence for the involvement of CD8+ T lymphocytes in sporozoite transport and host protection. Poult Sci. (1995) 74:1117–25. 10.3382/ps.0741117 7479488

[27] Tierney J Mulcahy G. Comparative development of Eimeria tenella (Apicomplexa) in host cells in vitro. Parasitol Res. (2003) 90:301–4. 10.1007/s00436-003-0846-1 12684886

[28] Long PL Reid WM. Guide for the diagnosis of coccidiosis in chickens. The University of Georgia College of agricultura experiment station research report. (1982), 404.

[29] Vetterling JM Doran DJ. Schizogony and gametogony in the life cycle of the poultry coccidium, *Eimeria acervulina* Tyzzer, 1929. J Parasitol. (1966) 12:1150–57. 10.2307/3276360 5926340

[30] Novilla MN Jeffers TK Griffing WJ White SL. A redescription of the life cycle of *Eimeria mitis* Tyzzer, 1929. J Protozool. (1987) 34: 87–92. 10.1111/j.1550-7408.1987.tb03139.x 3572845

[31] Dubey JP Jenkins MC. Re-evaluation of the life cycle of *Eimeria maxima* Tyzzer, 1929 in chickens (*Gallus domesticus*). Parasitology. (2018) 145:1051–58. 10.1017/S0031182017002153 29239290

[32] McDonald V Rose ME. *Eimeria tenella* and *E. necatrix*: a third generation of schizogony is an obligatory part of the developmental cycle. J Parasitol. (1987) 17:617–22. 10.2307/3282145 3598808

[33] Duffy CF Mathis GF Power RF. Effects of Natustat™ supplementation on performance, feed efficiency and intestinal lesion scores in broiler chickens challenged with *Eimeria acervulina*, Eimeria maxima and *Eimeria tenella*. Vet Parasitol. (2005) 130:185–90. 10.1016/j.vetpar.2005.03.041 15905033

[34] Ahmad TA El-Sayed BA El-Sayed LH. Development of immunization trials against *Eimeria* spp. Trials Vaccinol. (2016) 5:38–47. 10.1016/j.trivac.2016.02.00116230106

[35] Walker RA Ferguson DJ Miller CM Smith NC. Sex and Eimeria: a molecular perspective. Parasitology. (2013) 140:1701–17. 10.1017/S0031182013000838 23953058

[36] Tewari AK Maharana BR. Control of poultry coccidiosis: changing trends. J Parasit Dis. (2011) 35:10–7. 10.1007/s12639-011-0034-7 22654309 PMC3114971

[37] Lee DL Millard BJ. The structure and development of the macrogamete and oocyst of *Eimeria acervulina*. Parasitology. (1971) 62:31–34. 10.1017/S0031182000071262 5102453

[38] Scholtyseck E Mehlhorn H Hammond DM. Electron microscope studies of microgametogenesis in coccidia and related groups. Z Parasitenkd. (1972) 38:95–131. 10.1007/BF00329023 4622927

[39] You MJ. The comparative analysis of 618 infection pattern and oocyst output in *Eimeria tenella*, E. maxima and E acervulina in young broiler chicken *Vet World*. (2014) 7:542–7. 10.14202/vetworld.2014.542-547

[40] Waldenstedt L Elwinger K Lunden A Thebo P Uggla A. Sporulation of Eimeria maxima oocysts in litter with different moisture contents. Poult Sci. (2001) 80:1412–15. 10.1093/ps/80.10.1412 11599698

[41] Awais MM Akhtar M Iqbal Z Muhammad F Anwar MI. Seasonal prevalence of coccidiosis in industrial broiler chickens in Faisalabad, Punjab, Pakistan. Trop Anim Health Pro. (2011) 44:323–28. 10.1007/s11250-011-0024-x 22102015

[42] Venkateswara RP Raman M Gomathinayagam S. Sporulation dynamics of poultry Eimeria oocysts in Chennai. J Parasit Dis. (2015) 39:689–92. 10.1007/s12639-013-0403-5 26688635 PMC4675589

[43] Edgar SA. Sporulation of oocysts at specific temperatures and notes on the prepatent period of several species of avian coccidia. J Parasitol. (1955) 41:214–16. 10.2307/3273795 14368440

[44] Norton CC Chard MJ. The oocyst sporulation time of *Eimeria* species from the fowl. Parasitol. (1983) 86:193–8. 10.1017/S0031182000050368 6856329

[45] Reid WM Long PL. A diagnostic chart for nine species of fowl coccidian. Georgia Agric. exp. Stn. Tech. Bull. Editor Bowen N. B., Athen: College of Agriculture, University of Georgia (1979). p 5–24.

[46] Arabkhazaeli F Nabian S Modirsaneii M Mansoori B Rahbari S. Biopathologic characterization of three mixed poultry *Eimeria* spp. isolates. I Iran J Parasitol. (2011) 6:23. 22347310 PMC3279901

[47] Györke A Pop L Cozma V. Prevalence and distribution of *Eimeria* species in broiler chicken farms of different capacities. Parasite. (2013) 20:50. 10.1051/parasite/2013052 24309007 PMC3852269

[48] Kant V Singh P Verma PK Bais I Parmar MS Gopal A. Anticoccidial drugs used in the poultry: an overview. Sci Int. (2013) 1:261–65. 10.17311/sciintl.2013.261.26531152233

[49] Conway DP McKenzie ME. Poultry Coccidiosis: Diagnostic and Testing Procedures. London: John Wiley & Sons. (2007).

[50] Morris GM Woods WG Richards DG Gasser RB. Investigating a persistent coccidiosis problem on a commercial broiler–breeder farm utilising PCR-coupled capillary electrophoresis. Parasitol Res. (2007) 101:583–89. 10.1007/s00436-007-0516-9 17404757

[51] Cantacessi C Riddell S Morris GM Doran T Woods WG. Otranto, D, Gasser RB. Genetic characterization of three unique operational taxonomic units of Eimeria from chickens in Australia based on nuclear spacer ribosomal DNA. Vet Parasitol. (2008) 152:226–34. 10.1016/j.vetpar.2007.12.028 18243560

[52] Blake DP Vrba V Xia D Jatau ID Spiro S Nolan MJ Tomley FM. Genetic and biological characterisation of three cryptic *Eimeria* operational taxonomic units that infect chickens (*Gallus gallus domesticus*). Int J Parasitol. (2021) 51:621–34. 10.1016/j.ijpara.2020.12.004 33713650 PMC8186487

[53] Joyner LP Long PL. The specific characters of the *Eimeria*, with special reference to the coccidia of the fowl. Avian Pathol. (1974) 3:145–57. 10.1080/03079457409353827 18777269

[54] Williams RB. Effects of different infection rates on the oocyst production of *Eimeria acervulina* or *Eimeria tenella* in the chicken. Parasitology. (1973) 67:279–88. 10.1017/S0031182000046515 4761768

[55] Williams RB. Quantification of the crowding effect during infections with the seven *Eimeria* species of the domesticated fowl: its importance for experimental designs and the production of oocyst stocks. Int J Parasitol. (2001) 31: 1056–69. 10.1016/S0020-7519(01)00235-1 11429169

[56] Jenkins MC. Dubey, JP, Miska K, Fetterer R. Differences in fecundity of Eimeria maxima strains exhibiting different levels of pathogenicity in its avian host. Vet Parasitol. (2017) 236:1–6. 10.1016/j.vetpar.2017.01.009 28288751

[57] Lillehoj HS Lillehoj EP. Avian coccidiosis. A review of acquired intestinal immunity and vaccination strategies. Avian Dis. (2000) 44:408–25. 10.2307/1592556 10879922

[58] Lillehoj HS. Influence of inoculation dose, inoculation schedule, chicken age, and host genetics on disease susceptibility and development of resistance to *Eimeria tenella* infection. Avian Dis. (1988) 32:437–44. 10.2307/1590909 3264150

[59] Hadas G Mebrhatu G. Abebe T. Prevalence of poultry coccidiosis in Gondar town, North West Ethiopia Am Eurasian. J Agric Environ Sci. (2014) 9:129–35. 10.5829/idosi.aejsr.2014.9.5.86147

[60] Wondimu A Mesfin E Bayu Y. Prevalence of poultry coccidiosis and associated risk factors in intensive farming system of Gondar Town, Ethiopia. Vet Med Int. (2019) 2019:1–6. 10.1155/2019/5748690 32089814 PMC7012234

[61] Greenacre CB Morishita TY. Backyard Poultry Medicine and Surgery: A Guide for Veterinary Practitioners. Hoboken, NJ : John Wiley & Sons. (2021).

[62] Peek HW. Resistance to Anticoccidial Drugs: Alternative Strategies to Control Coccidiosis in Broilers, Doctoral dissertation, Utrecht University. (2010).

[63] Ontario Ministry of Agriculture. Managing Coccidiosis in My Poultry Flock. Availale online at: https://atrium.lib.uoguelph.ca/xmlui/bitstream/handle/10214/11932/ManagingCoccidiosisInMyPoultryFlock.pdf?sequence=3. (accessed July 2021).

[64] Carvalho FS Wenceslau AA Teixeira M Carneiro JAM Melo ADB Albuquerque GR. Diagnosis of Eimeria species using traditional and molecular methods in field studies. Vet Parasitol. (2011) 176:95–100. 10.1016/j.vetpar.2010.11.015 21167646

[65] Barrios MA Da Costa M Kimminau E Fuller L Clark S Pesti G. Relationship between broiler body weights, *Eimeria maxima* gross lesion scores, and microscores in three anticoccidial sensitivity tests. Avian Dis. (2017) 61:237–41. 10.1637/11518-102116-Reg.1 28665718

[66] Hauck R Carriosa M McCrea BA Dormitorio T Macklin KS. Evaluation of next-generation amplicon sequencing to identify *Eimeria* spp. of chickens. Avian Dis. (2019) 63:577–83. 10.1637/aviandiseases-D-19-00104 31865671

[67] Hinsu AT Thakkar JR Koringa PG Vrba V Jakhesara SJ Psifidi A. Illumina next generation sequencing for the analysis of *Eimeria* populations in commercial broilers and indigenous chickens. Front Vet Sci. (2018) 5:176. 10.3389/fvets.2018.00176 30105228 PMC6077195

[68] Yun CH Lillehoj HS Lillehoj EP. Intestinal immune responses to coccidiosis. Dev Comp Immunol. (2000) 24:303–24. 10.1016/S0145-305X(99)00080-4 10717295

[69] Ali H Naqvi F Tariq N. Prevalence of coccidiosis and its association with risk factors in poultry of Quetta, Pakistan. Asian J Appl Sci. (2014) 2:4.

[70] Adams C Vahl HA Veldman A. Interaction between nutrition and Eimeria acervulina infection in broiler chickens: development of an experimental infection model. Br J Nutr. (1996) 75:867–73. 10.1079/BJN19960192 8774231

[71] Assis RCL Luns FD Beletti ME Assis RL Nasser NM Faria ESM. Histomorphometry and macroscopic intestinal lesions in broilers infected with *Eimeria acervulina*. Vet Parasitol. (2010) 168:185–9. 10.1016/j.vetpar.2009.11.017 20080348

[72] Collier CT Hofacre CL Payne AM Anderson DB Kaiser P Mackie RI. Coccidia-induced mucogenesis promotes the onset of necrotic enteritis by supporting Clostridium perfringens growth. Vet Immunol Immunopathol. (2008) 122:104–15. 10.1016/j.vetimm.2007.10.014 18068809

[73] Al-Quraishy S Qasem MA Al-Shaebi EM Murshed M Mares MM Dkhil MA. Rumex nervosus changed the oxidative status of chicken caecum infected with *Eimeria tenella*. J King Saud Univ Sci. (2020) 32:2207-11. 10.1016/j.jksus.2020.02.034

[74] Pearson JP Brownlee IA. Structure and function of mucosal surfaces. Coloniz Mucos Surf. (2005) 13:3–16. 10.1371/journal.pone.0030287 22299034 PMC3267712

[75] Montagné L Piel C Lallès JP. Effect of diet on mucin kinetics and composition: nutrition and health implications. Nutr Rev. (2004) 62:105–14. 10.1111/j.1753-4887.2004.tb00031.x 15098857

[76] Sharma R Schumacher U. Morphometric analysis of intestinal mucins under different dietary conditions and gut flora in rats. Digest Dis Sci. (1995) 40:2532–39. 10.1007/BF02220438 8536508

[77] Meslin JC Fontaine N Andrieux C Variation of mucin distribution in the rat intestine, caecum and colon: effect of the bacterial flora. Comp Biochem Physiol Mol Integr Physiol. (1999) 123:235–39. 10.1016/S1095-6433(99)00056-2 10501018

[78] Al-Sheikhly F Al-Saieg A. Role of coccidia in the occurrence of necrotic enteritis of chickens. Avian Dis. (1980) 24:324–33. 10.2307/1589700 6254485

[79] Adhikari P Kiess A Adhikari R Jha R. An approach to alternative strategies to control avian coccidiosis and necrotic enteritis. J Appl Poultry Res. (2020) 29:515–34. 10.1016/j.japr.2019.11.005

[80] Ruff MD. Important parasites in poultry production systems. Vet Parasitol. (1999) 84:337–347. 10.1016/S0304-4017(99)00076-X 10456422

[81] Khater HF Ziam H Abbas A Abbas RZ Raza MA Hussain K. Avian coccidiosis: recent advances in alternative control strategies and vaccine development. Agrobiol Rec. (2020) 1:11–25. 10.47278/journal.abr/2020.00415839405

[82] Johnson J Reid WM. Anticoccidial drugs: lesion scoring techniques in battery and floor-pen experiments with chickens. Exp Parasitol. (1970) 28:30–6. 10.1016/0014-4894(70)90063-9 5459870

[83] Price KR. Use of live vaccines for coccidiosis control in replacement layer pullets. J Appl Poultry Res. (2012) 21:679–92. 10.3382/japr.2011-0048625638718

[84] Raman M Banu SS Gomathinayagam S Raj GD. Lesion scoring technique for assessing the virulence and pathogenicity of Indian field isolates of avian *Eimeria* species. Vet Arh. (2011) 81:259–71.

[85] Hodgson JN. Coccidiosis: Oocyst counting technique for coccidiostat evaluation. Exp Parasitol. (1970) 28:99–102. 10.1016/0014-4894(70)90073-1 5459879

[86] Bortoluzzi C Paras KL Applegate TJ Verocai GG. Comparison between Mcmaster and mini-FLOTAC methods for the enumeration of *Eimeria maxima* oocysts in poultry excreta. Vet Parasitol. (2018) 254:21–5. 10.1016/j.vetpar.2018.02.039 29657006

[87] Cringoli G Maurelli MP Levecke B Bosco A Vercruysse J Utzinger J. The Mini-FLOTAC technique for the diagnosis of helminth and protozoan infections in humans and animals. Nat Protoc. (2017) 12:1723. 10.1038/nprot.2017.067 28771238

[88] Silva LMR Vila-Viçosa MJM Maurelli MP Morgoglione ME Cortes HCE Cringoli G. Mini-FLOTAC for the diagnosis of Eimeria infection in goats: an alternative to McMaster. Small Ruminant Res. (2013) 114:280–83. 10.1016/j.smallrumres.2013.06.017

[89] Noel ML Scare JA Bellaw JL Nielsen MK. Accuracy and precision of mini-FLOTAC and McMaster techniques for determining equine strongyle egg counts. J Equine Vet Sci. (2017) 48:182–87. 10.1016/j.jevs.2016.09.006

[90] Daş G Klauser S Stehr M Tuchscherer A. Metges CC. Accuracy and precision of McMaster and Mini-FLOTAC egg counting techniques using egg-spiked faeces of chickens and two different flotation fluids. Vet Parasitol. (2020) 283:109158. 10.1016/j.vetpar.2020.109158 32544762

[91] You MJ. Detection of four important *Eimeria* species by multiplex PCR in a single assay. Parasitol Int. (2014) 63:527–32. 10.1016/j.parint.2014.01.006 24495953

[92] Hamidinejat H Shapouri MS Mayahi M Borujeni MP. Characterization of *Eimeria* species in commercial broilers by PCR based on ITS1 regions of rDNA. Iran J Parasitol. (2010) 5:48–54. 22347266 PMC3279851

[93] Kumar S Garg R Moftah A Clark EL Macdonald SE Chaudhry AS. An optimised protocol for molecular identification of *Eimeria* from chickens. Vet Parasitol. (2014) 199:24–31. 10.1016/j.vetpar.2013.09.026 24138724 PMC3858809

[94] Tang X Huang G Liu X El-Ashram S Tao G Lu C. An optimized DNA extraction method for molecular identification of coccidian species. Parasitol Res. (2018) 117:655–64. 10.1007/s00436-017-5683-8 29396674

[95] Shirley MW Bumstead N. Intra-specific variation within Eimeria tenella detected by the random amplification of polymorphic DNA. Parasitol Res. (1994) 80:346–51. 10.1007/BF02351878 8073024

[96] Fernandez S Katsuyama AM Kashiwabara AY Madeira AMB Durham AM Gruber A. Characterization of SCAR markers of *Eimeria* spp. of domestic fowl and construction of a public relational database (The Eimeria SCARdb). FEMS Microbiol Lett. (2004) 238:183–88. 10.1016/j.femsle.2004.07.034 15336420

[97] Shu-San L Lik-Sin L Efendi NA Blake DP Kawazu SI Wan KL. Comparison of molecular methods for the detection of *Eimeria* in domestic chickens in Malaysia. Sains Malays. (2019) 48:1425–32. 10.17576/jsm-2019-4807-11

[98] Barkway CP Pocock RL Vrba V Blake DP. Loop-mediated isothermal amplification (LAMP) assays for the species-specific detection of *Eimeria* that infect chickens. BMC Vet Res. (2011) 7:1–8. 10.1186/1746-6148-7-67 22053893 PMC3217895

[99] Moraes JC França M Sartor AA Bellato V de Moura AB Magalhães MDLB. Prevalence of *Eimeria* spp. in broilers by multiplex PCR in the southern region of Brazil on two hundred and fifty farms. Avian Dis. (2015) 59:277–81. 10.1637/10989-112014-Reg 26473679

[100] Clark EL Macdonald SE Thenmozhi V Kundu K Garg R Kumar S. Cryptic *Eimeria* genotypes are common across the southern but not northern hemisphere. Int J Parasitol. (2016) 46:537–44. 10.1016/j.ijpara.2016.05.006 27368611 PMC4978698

[101] Chapman HD Jeffers TK Williams RB. Forty years of monesin for the control of coccidiosis in poultry. Poult Sci J. (2010) 89:1788–801. 10.3382/ps.2010-00931 20709963

[102] Chapman HD. A landmark contribution to poultry science-Prophylactic control of coccidiosis in poultry. Poult Sci. (2009) 88:813–15. 10.3382/ps.2008-00316 19276426

[103] Poultry Health Today. Nearly 60% of US broilers now raised without antibiotics, but that number may have peaked. (2020). Available online at: https://poultryhealthtoday.com/nearly-60-of-usbbroilers-now-raised-without-antibiotics-but-that-number-may-have-peaked/?utm_source=Poultry+Health+Today+Newsletter&utm_campaign=daab24c737-AAAP_antimicrobial_stewardship_PHT_1_8_2018_COPY_0&utm_medium=email&utm_term=0_5ac605299a-daab24c737-315439401. (accessed Sept 2021).

[104] Chapman HD Jeffers TK. Vaccination of chickens against coccidiosis ameliorates drug resistance in commercial poultry production. Int J Parasitol Drugs Drug Resist. (2014) 4:214–17. 10.1016/j.ijpddr.2014.10.002 25516830 PMC4266793

[105] Witcombe DM Smith NC Hemphill A. Strategies for anti-coccidial prophylaxis. Parasitology. (2014) 141:1379–89. 10.1017/S0031182014000195 24534138

[106] Ilender. Salincarb®. (2021). Available online at: https://www.ilendercorp.com/productos/#/salinocarb/197/112/1/15. (accessed October 22, 2021).

[107] Zoetis. Gromax® (2013). Available online at: https://www.zoetisus.com/_locale-assets/poultry/poultry-literature-library/apac-en/gromax_productprofile_zp130181-a_apac-en_zoetis.pdf. (accessed October 22, 2021).

[108] Phibro- animal health corporation. Aviax ®Plus (2018). Available online at: https://phibrosaludanimal.com/news/descarga-aviax-plus/folletos/FOLL-Aviax-Plus-brochure-Digital.pdf. (accessed October 22, 2021).

[109] Huvepharma. Monimax ®. (2020). Available online at: https://www.huvepharma.com/news/article/unique-new-product-demonstrates-efficacy-and-performance//. (accessed October 22, 2021).

[110] Impextraco. Lerberk®. (2019). Available online at: https://www.impextraco.com/products/enhancing-animals/xtra-performance-xp-anticoccidials/coccidialsolution. (accessed October 22, 2021).

[111] Feed additive Compendium. Narasin/Nicarbazin- Chickens (2018). p. 264.

[112] Rose ME Hesketh P. Immunity to coccidiosis: stages of the life-cycle of *Eimeria maxima* which induce, and are affected by, the response of the host. Parasitology. (1976) 73:25–37. 10.1017/S0031182000051295 967527

[113] Dalloul RA Lillehoj HS. Poultry coccidiosis: recent advancements in control measures and vaccine development. Exp Rev Vaccines. (2006) 5:143–63. 10.1586/14760584.5.1.143 16451116

[114] Rose ME Wakelin D Hesketh P. Eimeria vermiformis: differences in the course of primary infection can be correlated with lymphocyte responsiveness in the BALB/c and C57BL/6 mouse, Mus musculus. Exp Parasitol. (1990) 71:276–83. 10.1016/0014-4894(90)90032-8 2209786

[115] Rose ME Hesketh P Rothwell L Gramzinski RA. T-cell receptor gamma–delta lymphocytes and Eimeria vermiformis infection. Infect Immun. (1996) 64:4854–858. 10.1128/iai.64.11.4854-4858.1996 8890252 PMC174458

[116] Lillehoj HS Choi KD. Recombinant chicken interferongamma- mediated inhibition of *Eimeria tenella* development in vitro and reduction of oocyst production and body weight loss following *Eimeria acervulina* challenge infection. Avian Dis. (1998) 42:307–14. 10.2307/1592481 9645322

[117] Fatoba AJ Adeleke MA. Diagnosis and control of chicken coccidiosis: a recent update. J Parasitic Dis. (2018) 42:483–93. 10.1007/s12639-018-1048-1 30538344 PMC6261147

[118] Lillehoj HS. Effects of immunosuppression on avian coccidiosis: cyclosporin a but not hormonal bursectomy abrogates host protective immunity. Infect Immun. (1987) 55:1616–21. 10.1128/iai.55.7.1616-1621.1987 3496277 PMC260567

[119] López-Osorio S Chaparro-Gutiérrez JJ Gómez-Osorio LM. Overview of poultry eimeria life cycle and host-parasite interactions. Front Vet Sci. (2020) 7:384. 10.3389/fvets.2020.00384 32714951 PMC7351014

[120] Shirley MW Smith AL Tomley FM. The biology of avian *Eimeria* with an emphasis on their control by vaccination. Adv Parasitol. (2005) 60:285–330. 10.1016/S0065-308X(05)60005-X 16230106

[121] Blake DP Marugan-Hernandez V Tomley FM. Spotlight on avian pathology: *Eimeria* and the disease coccidiosis. Avian Pathol. (2021) 50:209–13. 10.1080/03079457.2021.1912288 33823695

[122] Blake DP Pastor-Fernández I Nolan MJ Tomley FM. Recombinant anticoccidial vaccines-a cup half full?. Infect Genet Evol. (2017) 55:358–65. 10.1016/j.meegid.2017.10.009 29017798

[123] Blake DP Knox J Dehaeck B Huntington B Rathinam T Ravipati V. Re-calculating the cost of coccidiosis in chickens. VetRes. (2020) 51:1–14. 10.1186/s13567-020-00837-2 32928271 PMC7488756

[124] Jeffers TK. Attenuation of *Eimeria tenella* through selection for precociousness. J Parasitol. (1975) 75:1083–90. 10.2307/3279381 1195070

[125] Fetterer RH Jenkins MC Miska KB Barfield RC. Evaluation of an experimental irradiated oocyst vaccine to protect broiler chicks against avian coccidiosis. Avian Dis. (2014) 58:391–7. 10.1637/10679-092613-Reg.1 25518433

[126] Gadelhaq SM Arafa WM Dahshan AHM Abolhadid SM. Using of diclazuril in attenuation of *Eimeria* species for induction of protective immunity against coccidiosis in layer chicks. Assiut Vet Med J. (2017) 63:101–08. 10.21608/avmj.2017.170969

[127] Shirley MW Bedrnik P. Live attenuated vaccines against avian coccidiosis: success with precocious and egg-adapted lines of Eimeria Parasitol. Today. (1997) 13:481–84. 10.1016/S0169-4758(97)01153-8 15275137

[128] Sharman PA Smith NC Wallach MG Katrib M. Chasing the golden egg: vaccination against poultry coccidiosis. Parasite Immunol. (2010) 32:590–98. 10.1111/j.1365-3024.2010.01209.x 20626814

[129] Hoelzer K Bielke L Blake DP Cox E Cutting SM Devriendt B. Vaccines as alternatives to antibiotics for food producing animals. Part 2: new approaches and potential solutions. Vet Res. (2018) 49:70. 10.1186/s13567-018-0561-7 30060759 PMC6066917

[130] Pastor-Fernández I Kim S Marugán-Hernández V Soutter F Tomley FM Blake DP. Vaccination with transgenic Eimeria tenella expressing Eimeria maxima AMA1 and IMP1 confers partial protection against high-level E. *maxima* challenge in a broiler model of coccidiosis. Parasit Vect. (2020) 13:1–12. 10.1186/s13071-020-04210-2 32650837 PMC7350274

[131] Sharaban H Seadawy M El-khayat F El-Gohary AEG. Evaluation of coccidiosis vaccines in chicken. Kafr El-Sheikh Vet Med J. (2021) 19:13–19. 10.21608/kvmj.2021.73171.1018

[132] Kimminau EA. Duong T. Longitudinal response of commercial broiler operations to bio-shuttle administration. J Appl Poult Res. (2019) 28:1389–97. 10.3382/japr/pfz092

[133] Abbas RZ Iqbal Z Khan A Sindhu ZUD Khan JA Khan MN. Options for integrated strategies for the control of avian coccidiosis. Int J Agric Biol. (2012) 14:1014–20.

[134] Cervantes HM. Antibiotic-free poultry production: is it sustainable? J Appl Poult Res. (2015) 24:91–7. 10.3382/japr/pfv006

[135] Chapman HD Barta JR Blake D Gruber A Jenkins M Smith NC. A selective review of advances in coccidiosis research. Adv Parasit. (2013) 83:93–171. 10.1016/B978-0-12-407705-8.00002-1 23876872

[136] Remmal A Achahbar S Bouddine L Chami N Chami F. In vitro destruction of *Eimeria* oocysts by essential oils. Vet Parasitol. (2011) 182:121–26. 10.1016/j.vetpar.2011.06.002 21726944

[137] Scheurer W Spring P Maertens L. Effect of 3 dietary phytogenic products on production performance and coccidiosis in challenged broiler chickens. J Appl Poult Res. (2013) 22:591–9. 10.3382/japr.2013-00726

[138] Li J Xing T Wang L Tao J. Liu, Z. Inhibitory effect of S-nitroso-glutathione on Eimeria tenella oocysts was mainly limited to the early stages of sporogony. Vet Parasitol. (2010) 173:64–9. 10.1016/j.vetpar.2010.06.022 20638798

[139] Rathinam T Gadde U Chapman HD. Sericea lespedeza has no anticoccidial effect when included in the diet of chickens infected with three species of *Eimeria*. Vet Parasitol. (2014) 202:265–9. 10.1016/j.vetpar.2014.01.017 24594212

[140] Kadykalo S Roberts T Thompson M Wilson J Lang M Espeisse O. The value of anticoccidials for sustainable global poultry production. Int J Antimicrob Agents. (2018) 51:304–10. 10.1016/j.ijantimicag.2017.09.004 28935212

[141] Idris M Abbas RZ Masood S Rehman T Farooq U Babar W. The potential of antioxidant rich essential oils against avian coccidiosis. Worlds Poult Sci J. (2017) 73:89–104. 10.1017/S004393391600078730886898

[142] Mesa C Gómez-Osorio LM López-Osorio S Williams SM. Chaparro-Gutiérrez JJ. Survey of coccidia on commercial broiler farms in Colombia: frequency of Eimeria species, anticoccidial sensitivity, and histopathology. Poult Sci J. (2021) 100:101239. 10.1016/j.psj.2021.101239 34214749 PMC8255230

[143] Mattiello R Boviez JD McDougald LR. Eimeria brunetti and Eimeria necatrix in chickens of Argentina and confirmation of seven species of Eimeria. Avian Dis. (2000) 44:711–4. 10.2307/1593117 11007025

[144] Prakashbabu BC Thenmozhi V Limon G Kundu K Kumar S Garg R. *Eimeria* species occurrence varies between geographic regions and poultry production systems and may influence parasite genetic diversity. Vet Parasitol. (2017) 233:62–72. 10.1016/j.vetpar.2016.12.003 28043390 PMC5239766

[145] Sun XM Pang W Jia T Yan WC He G Hao LL. Prevalence of *Eimeria* species in broilers with subclinical signs from fifty farms. Avian Dis. (2009) 53:301–5. 10.1637/8379-061708-Resnote.1 19630240

[146] Lee BH Kim WH Jeong J Yoo J Kwon YK Jung BY Min W. Prevalence and cross-immunity of *Eimeria* species on Korean chicken farms. J Vet Med Sci. (2010) 72:985–89. 10.1292/jvms.09-0517 20234110

[147] Gharekhani J Sadeghi-Dehkordi Z. Bahrami M. Prevalence of coccidiosis in broiler chicken farms in Western Iran. J Vet Med. (2014) 2014:1–5. 10.1155/2014/980604 26464948 PMC4590881

[148] Kaboudi K Umar S Munir MT. Prevalence of coccidiosis in free-range chicken in Sidi Thabet, Tunisia. Scientifica. (2016) 2016:1–6. 10.1155/2016/7075195 27213084 PMC4860226

